# Comparative analysis of different molecular and serological methods for detection of *Xylella fastidiosa* in blueberry

**DOI:** 10.1371/journal.pone.0221903

**Published:** 2019-09-03

**Authors:** Sumyya Waliullah, Owen Hudson, Jonathan E. Oliver, Phillip M. Brannen, Pingsheng Ji, Md Emran Ali

**Affiliations:** 1 Department of Plant Pathology, University of Georgia, Tifton, GA, United States of America; 2 Department of Plant Pathology, University of Georgia, Athens, GA, United States of America; Panstwowy Instytut Weterynaryjny - Panstwowy Instytut Badawczy w Pulawach, POLAND

## Abstract

Bacterial leaf scorch, caused by *Xylella fastidiosa*, is a major threat to blueberry production in the southeastern United States. Management of this devastating disease is challenging and often requires early detection of the pathogen to reduce major loss. There are several different molecular and serological detection methods available to identify the pathogen. Knowing the efficiency and suitability of these detection techniques for application in both field and laboratory conditions is important when selecting the appropriate detection tool. Here, we compared the efficiency and the functionality of four different molecular detection techniques (PCR, real-time PCR, LAMP and AmplifyRP® Acceler8™) and one serological detection technique (DAS-ELISA). The most sensitive method was found to be real-time PCR with the detection limit of 25 fg of DNA molecules per reaction (≈9 genome copies), followed by LAMP at 250 fg per reaction (≈90 copies), AmplifyRP® Acceler8™ at 1 pg per reaction (≈350 copies), conventional PCR with nearly 1.25 pg per reaction (≈ 440 copies) and DAS-ELISA with 1x10^5^ cfu/mL of *Xylella fastidiosa*. Validation between assays with 10 experimental samples gave consistent results beyond the variation of the detection limit. Considering robustness, portability, and cost, LAMP and AmplifyRP® Acceler8™ were not only the fastest methods but also portable to the field and didn’t require any skilled labor to carry out. Among those two, AmplifyRP® Acceler8™ was faster but more expensive and less sensitive than LAMP. On the other hand, real-time PCR was the most sensitive assay and required comparatively lesser time than C-PCR and DAS-ELISA, which were the least sensitive assays in this study, but all three assays are not portable and needed skilled labor to proceed. These findings should enable growers, agents, and diagnosticians to make informed decisions regarding the selection of an appropriate diagnostic tool for *X*. *fastidiosa* on blueberry.

## Introduction

*Xylella fastidiosa* is a xylem-limited, gram-negative, fastidious bacterium that causes economically important diseases in many plants including citrus, grapevine, almond, peach, and pear [1]. Outbreaks of new diseases caused by this bacterium have become a worldwide threat. In the U.S., the bacterium was first reported to cause disease in grapes (*Vitis vinifera* L.) in Southern California in 1892 [2] and was isolated from grapevines with Pierce's disease (PD) [3]. It was later reported in several parts of California and other states including Texas, Florida, and Georgia [4–9]. In 2005, a new disorder caused by *X*. *fastidiosa*, Bacterial Leaf Scorch, appeared in southern highbush blueberry cultivars (*Vaccinium corymbosum* interspecific hybrids) with high market value in the state of Georgia [9, 10]. The bacteria can be spread through both vegetative propagation and via insect transmission [4, 11, 12], with initial symptoms of marginal leaf scorch (burn) of older leaves, severely reduced vegetative growth with reduced numbers of flower buds, and yellowed stems and twigs [9]. Leaf drop occurs in the later stage of infection and eventually leads to plant death [9]. Management of bacterial leaf scorch is challenging and only a few control options are available for this pathogen. Among these options, the prompt removal of infected plants is one of the key strategies, as diseases caused by *Xylella* spp. can spread from ~8,000 ha to ~23,000 ha within just a few months [11]. Early detection of this pathogen can aid in decreasing major crop loss and can further prevent the spread of the disease [13].

In contrast, conventional field and laboratory-based approaches such as isolation or culturing of the bacterium on agar media [14, 15] to detect and identify *X*. *fastidiosa*, serological and especially molecular-based detection assays are suitable for large numbers of samples and have greater specificity and sensitivity. Along with the detection of *X*. *fastidiosa* by serological methods like Enzyme-linked immunosorbent assay (ELISA) [16] or double antibody sandwich (DAS)-ELISA [14], western-blotting [17] and immunofluorescence [18], several polymerase chain reaction (PCR) -based molecular detection methods are also being used widely including conventional PCR [19, 20], TaqMan probe-based singleplex and multiplex real-time PCR for species-specific and universal detection [21–23], SYBR® Green-based real-time PCR and reverse transcriptase quantitative PCR (RT-qPCR) [24, 25]. PCR derivatives like Loop-mediated isothermal amplification (LAMP) have also been used recently to detect the pathogen [26], a method which is based on isothermal amplification of nucleic acids and can be performed in a heat block or water bath without any need for a thermocycler. LAMP has recently been utilized in the detection of plant pathogens and is a promising substitute for PCR-based detection systems due to its high sensitivity, accuracy, and ability to provide quicker results [26, 27]. It can be utilized for on-site detection of the pathogen with results visualized by the colorimetric SYBR green reaction at the endpoint [28], by hydroxy naphthol blue (HNB) which develops a purple color in the presence of Mg^2+^ [27], or by changed yellow color of the pH-sensitive dye phenol red [29]. In addition, a recent recombinase-polymerase amplification (RPA) based end-product detection technology that can be used for onsite detection with high sensitivity and rapidity has been developed by Agdia® Inc., i.e. the end-product detection technology AmplifyRP® Acceler8® and real-time detection AmplifyRP® XRT [30]. Although all these molecular and serological methods are widely used from the laboratory to field in order to detect pathogens, there are limited reports available to show the comparison amongst them. Previously, Loconsole *et al*. [31] diagnosed *X*. *fastidiosa* from olive trees affected by Olive Quick Decline Syndrome (OQDS) using C-PCR and ELISA assays and showed comparison between the two techniques by interlaboratory ring-test. In another report, Harper et al. [26] developed a new LAMP and real-time PCR assay targeting the 16s rRNA processing protein which was superior to the existing LAMP and real-time PCR assays and compared the two assays by checking their detection limits. Despite those studies, there are still no comparative studies to determine which detection method is fastest, most economical, most accurate, or has transferability between laboratory and on-site detection to detect disease-causing agents. In this study, we used DNA from a pure culture of *X*. *fastidiosa* and tissue from infected blueberry plants to compare the functionality of C-PCR, real-time PCR, LAMP, ELISA (Enzyme-Linked immunosorbent assay), and Agdia® RPA end-product detection technology AmplifyRP ® Acceler8®. Our goal was to provide growers, farmers, and diagnosticians with research based data to allow them to make informed decisions regarding the most effective diagnostic techniques for bacterial leaf scorch disease of blueberry.

## Methods

### Plant samples and tissue preparation

Blueberry plant samples infected with *X*. *fastidiosa* were collected from green house and field to use in this study for bacterial detection. The cultivar used in field and greenhouse studies was southern highbush blueberry cultivar ‘Rebel’. The greenhouse plants were grown and inoculated with an isolate of *X*. *fastidiosa* according to the methods described by Oliver *et al*. [32] as adapted from Chang *et al*. [9]. The isolate was *X*. *fastidiosa* subsp. *multiplex* originally isolated from naturally infected ‘Rebel’ blueberry plants in Bacon County, Georgia. Collected samples from field were naturally infected ‘Rabel’ blueberry plants from Appling County, Georgia. Plant samples used for serological and molecular detection was 5–6” long internodes with 10 to 12 mature leaves Mature leave samples were surface sterilized with 5% sodium hypochlorite solution before each assay.

### DNA extraction

As *X*. *fastidiosa* colonize within the xylem network of plants, so for DNA extraction from infected leaves leaf petioles and midribs were taken. For this purpose, 1-cm long pieces were excised from 8–10 leaf petioles and midribs were flash frozen in liquid nitrogen and pulverized using a mortar and pestle. For experimental sample analysis, ten total samples were processed, 5 from infected field plants and 5 from infected greenhouse plants. Total DNA was extracted (10 replications) using the DNeasy Plant Kit (Qiagen, Valencia, CA) from 150 mg of homogenized tissue with a slight modification to the kit protocol. For detection of *X*. *fastidiosa* and sensitivity analysis of C-PCR, qPCR, LAMP and AmplifyRP® Acceler8™ assays, aliquots of 200 ng/μl *X*. *fastidiosa* DNA prepared following extraction from pure bacterial culture were used. These were mixed with uninfected healthy blueberry DNA samples with the ratio of 1:1 mixture (where the final concentration of DNA for Xf or blueberry was 100 ng/μl) to mimic the composition of DNA extracts from infected blueberry tissues and then serially diluted to 10, 1, 0.1, 0.01, 0.005, 0.0025, 0.00125, 0.001, 0.0005, 0.00025, 0.000125, 0.0001, 0.00005, 0.000025, 0.0000125 and 0.00001 ng/μl. Cultures were originally isolated from *X*. *fastidiosa*-infected blueberry collected in Bacon County, Georgia. Isolations were carried out on periwinkle wilt media according to the method described by Davis *et al*. [33]. Genomic DNA of pure culture of *X*. *fastidiosa* was extracted using the Qiagen Genomic DNA Isolation Kit (Valencia, CA). Total DNA yield and purity was estimated by measuring OD 260nm and OD 260nm/280nm with a NanoDrop spectrophotometer (NANODROP LITE, Thermo Scientific, Wilmington, DE). On-site DNA extraction was also carried out of *X*. *fastidiosa* from infected leaf in green house using a QuickPick™ SML Plant DNA kit and PickPen 1-M (Bio-Nobile, Turku, Finland), following the manufacturer’s instructions with some minor modifications. The eluted DNA was used for downstream application for on-site detection of pathogen using LAMP assay.

### Conventional PCR (C-PCR)

For C-PCR assay sensitivity analysis and to detect *X*. *fastidiosa* from blueberry leaf samples, primer set RST 31/33 was used in this work have previously been widely used for detection of *X*. *fastidiosa* and target a conserved genomic region of the RNA polymerase sigma factor (Table 1). Three other C-PCR primer sets (Table 1) were used only to determine the sensitivity of the assay. C-PCR reactions were performed on a thermocycler (Biorad-96 well T100™, Bio-rad, Hercules, CA) using EconoTaq PLUS GREEN 2X Master Mix (Lucigen, Madison, WI) based on the manufacturer’s suggested protocol. For C-PCR sensitivity analysis, each reaction contained 1 μl from each serially diluted DNA with 0.3 μM of each forward and reverse primer (Table 1), 10 μl of 2X Econotaq master mix (Lucigen, Madison, WI), and deionized PCR grade water to make a final volume of 20 μl. For the analysis of the experimental samples from greenhouse and field, 50 ng of DNA was used in each reaction mixture. For both analyses, C-PCR conditions for RST 31/33 was as follows: an initial denaturation step at 95°C for 2 min followed by 40 cycles of 30 sec at 95°C, 30 sec at 58°C, 45 sec at 72°C and final extension at 72°C for 5 min. For other C-PCR primers, the conditions were as follows: for S-S-X.fas-0838-a-S-21/S-S-X.fas-1439-a-A-19, initial denaturation step at 95°C for 3 min followed by 40 cycles of 30 sec at 94°C, 30 sec at 55°C, 40 sec at 72°C and final extension at 72°C for 5 min; for FXYgyr499/RXYgyr907, initial denaturation was at 94°C for 3 min followed by 40 cycles of 30 sec at 94°C, 30 sec at 55°C, 1 min at 72°C and final extension at 72°C for 5 min and for HL5/HL6, initial denaturation temperature was 95°C for 2 min followed by 40 cycles of 10 sec at 95°C, 15 sec at 60°C, 30 sec at 72°C and final extension at 72°C for 5 min.C-PCR products were checked on 1.0% Tris-borate-EDTA (TBE) agarose gel. Samples were considered PCR positive when the DNA band of the expected size (733 bp for RST 31/RST 33, 603 bp for S-S-X.fas-0838-a-S-21/S-S-X.fas-1439-a-A-19, 428 bp for FXYgyr499/RXYgyr907 and 221 bp for HL5/HL6) was clearly visualized after electrophoresis.

**Table 1:** Oligonucleotide sequences used for C-PCR, real-time PCR and LAMP.

Assay	Primers name	Target gene Name	Sequence (5’-3’)	References
PCR	RNA polymerase sigma factor	RST-31F	GCGTTAATTTTCGAAGTGATTCGATTGC	Minsavage *et al*., 1994
		RST-33R	CACCATTCGTATCCCGGTG	
	16S rRNA	S-S-X.fas-0838-a-S-21	GCAAATTGGCACTCAGTATCG	Rodriguez et al., 2003
		S-S-X.fas-1439-a-A-19	CTCCTCGCGGTTAAGCTAC	
	gyrB	FXYgyr499	CAGTTAGGGGTGTCAGCG	Rodriguez et al., 2003
		RXYgyr907	CTCAATGTAATTACCCAAGGT	
	Internal transcriber spacers (ITS)	HL5	AAGGCAATAAACGCGCACTA	Francis et al., 2006
		HL6	GGTTTTGCTGACTGGCAACA	
Real-time PCR	16srRNA processing protein	XF-F	CACGGCTGGTAACGGAAGA	Harper et al., 2010
		XF-R	GGGTTGCGTGGTGAAATCAAG	
LAMP	16srRNA processing protein	XF-F3	CCGTTGGAAAACAGATGGGA	Harper et al., 2010
		XF-B3	GAGACTGGCAAGCGTTTGA	
		XF-FIP	ACCCCGACGAGTATTACTGGGTTTTTCGCTACCGAGAACCACAC	
		XF-BIP	GCGCTGCGTGGCACATAGATTTTTGCAACCTTTCCTGGCATCAA	
		XF-LF	TGCAAGTACACACCCTTGAAG	
		XF-LB	TTCCGTACCACAGATCGCT	

### Real-time PCR

The real-time PCR assay was performed in a Cepheid smart cycler II (Sunnyvale, CA) using iQ™ SYBR Green Supermix (BioRad Laboratories Inc., Hercules, CA) in a 25 μl reaction using previously reported primers (16s rRNA processing protein, Table 1), according to manufacturer’s protocol. Each reaction mixture contained 12.5 μl of Biorad iQ™ SYBR Green Supermix, 0.3 μM each of the forward and reverse primer (Table 1), 50 ng of experimental DNA sample, and deionized PCR grade water for a final volume of 25 μl. Optimal thermocycling conditions were used for all reactions beginning with an initial denaturing step of 95°C for 120 sec with optics off, followed by 40 cycles of 95°C for 10 sec with optics off and 60°C for 40 sec with optics on and a temperature ramp at 0.2°C/sec for the entire protocol. All samples were amplified in triplicate and each run contained one positive control of the DNA extracted from *X*. *fastidiosa* pure culture, one negative control of healthy blueberry plant DNA, and deionized water as template control. Data was exported from the SmartCycler to calculate mean Ct values and standard error mean (SEM). To check the sensitivity of the assay and calculate copy number of the experimental *X*. *fastidiosa* infected blueberry tissue samples, a standard curve was prepared according to Harper *et al*. and Francis *et al*. [26, 34] from the serial dilutions of the *X*. *fastidiosa* DNA extracted from the pure culture of the pathogen at concentrations ranging from 10 fg/μl to 100 ng/μl (see methods). Standard linear regression (y = -3.4491x + 38.833, R^2^ = 0.9915, S1 Fig) was obtained by plotting the log concentration of *X*. *fastidiosa* copies at the different dilutions (Y) versus the mean Ct values (X). The efficiency (E) of the real-time PCR reaction was 95% for *X*. *fastidiosa* 16s rRNA processing protein (XF-F/R) which was calculated with the formula E = [10(−1/slope)–1] [25] (S1 Fig). Cycle threshold values >38 were considered as negatives in this study.

### LAMP assay

To detect the pathogen using LAMP amplification, LavaLAMP™ DNA Master Mix (Lucigen, WI, USA) and WarmStart® Colorimetric LAMP 2X Master Mix (New England Bio Labs Ltd., UK) which utilizes pH sensitive phenol red as an end-point indicator, was used according to manufacturer’s instruction. Each reaction mixture used in this study contained 12.5 μl 2X master mix, 2.5 μl of primer mix, 50 ng of *X*. *fastidiosa* experimental DNA samples and 1 μl samples from serially diluted pure culture (previously described in methods) for sensitivity analysis of the assay for *X*. *fastidiosa* detection with the rest were filled with DNase/RNase free PCR certified water (TEKNOVA, Hollister, USA) to a final volume of 25 μl. For LavaLAMP™ master mix (Lucigen), the primer mixture was comprised of 2 μM of each F3/B3, 8 μM of each LF/LB and 16 μM of each FIP/BIP primers, and for WarmStart® Colorimetric 2X Master Mix (NEB), 2 μM of each F3/B3, 4 μM of each LF/LB and 16 μM of each FIP/BIP primers were used (Table 1). All reactions were performed in 0.2-ml micro-tubes in a thermocycler (Biorad-96 well T100™, Bio-rad, Hercules, CA) with an amplification step at 70°C for 45 min and hold for 2 min at 4°C to stop the reaction. Each run contained DNA extract from pure *X*. *fastidiosa* culture as positive control and PCR grade water as negative control instead of DNA. LAMP PCR amplification products using LavaLAMP™ master mix (Lucigen) were evaluated by electrophoresis on 1.5% Tris-borate-EDTA (TBE) agarose gel stained with Gelgreen and visualized under ultraviolet (UV) light by Biorad molecular imager Gel Doc™ XR+ with image lab™ software (Biorad, Hercules, CA). Finally, the amplified end product from WarmStart® Colorimetric LAMP 2X Master Mix (NEB) was assessed visually with the naked eye.

### Double antibody sandwich ELISA (DAS-ELISA)

To check the sensitivity of DAS-ELISA (Agdia®, Inc. Elkhart, IN) for *X*. *fastidiosa* detection a known concentration of the pathogen was added to the sample buffer in the early stages of the extraction procedure. Suspensions of bacteria from 4 to 5-day old cultures were standardized in buffer to get cell concentrations ranging from 10^9^ to 10^1^ cfu/ml by checking optical density with a spectrophotometer (Thermo Scientific™ NanoDrop™ One^c^, Wilmington, DE) at OD 600 nm. Then, 100 μl suspensions were homogenized in a shear-resistant 1.5 ml safe lock microcentrifuge tubes with steel beads (Scientific Instrument Services inc., Ringoes, NJ) with a Bullet-Blender® Homogenizer (Scientific Instrument Services inc., Ringoes, NJ) containing healthy blueberry leaf petioles and midveins with general extraction buffer (Agdia®, Inc. Elkhart, IN) to make a final volume of 1.0 ml. For experimental samples, before starting tissue extraction for ELISA, samples (leaf petioles and mid-veins) were surface sterilized with 0.8% NaOCl solution for 3 min followed by rinsing three times with sterilized water (5 min/rinse). Then, sterilized tissues were cut into 1-cm^2^ small pieces using a sterilized razor blade and 0.3 grams of those were placed in a sterilized mortar with 3 ml of general extraction buffer (Agdia®, Inc. Elkhart, IN). The tissue was then crushed with a pestle at room temperature. Next, 100 μl of the pure culture and extracted sap with buffer solution were loaded into a 96-well microtiter plate pre-coated with peroxidase enzyme conjugate diluted 1:200 in 1xMRS diluent coating buffer (Xf. PathoScreen Kit, Agdia®, Inc.). The rest of the steps proceeded according to the manufacturer’s instruction (Agdia®, Inc.). Absorbance was measured with a plate reader (Multiskan® EX, Thermo Scientific, Vantaa, Finland) at 405 nm after 20 min of incubation at room temperature, and by visualizing their color development. The test plate included one positive control and a negative control (buffer only). Each sample was run in triplicate. Samples were determined to be positive if the absorbance was three times greater than the mean absorbance of healthy control samples [31].

### Xf AmplifyRP® Acceler8™ end point detection

To test the sensitivity of the recombinase-polymerase-amplification (RPA) technology based AmplifyRP® Acceler8™ end-point detection assay (Agdia® Inc.) using AmplifyRP® XRT+ for Xf kit (Agdia® Inc. Cat# XCS 34501/0048), serially diluted DNA samples from pure *X*. *fastidiosa* cultures were used ranging from 10 fg/μl to 100 ng/μl (see methods). To determine the end point for the assay, 1 μl from each of the serially diluted DNA samples were mixed with 24 μl of the pellet diluent buffer1 (PD1) into a 1.5-ml microcentrifuge tube. On the other hand, for experimental samples, DNA samples were diluted in PD1 buffer to make the final concentration of 50 ng per reaction with a final volume of 25μl [35]. Next, both of the 25 μl DNA samples with PD1 buffer from the endpoint detection assay and experimental samples were loaded into the 0.2-ml reaction pellet tubes and incubated for 20 min in the AmplifyRP® portable heat block (Agdia® Inc., Cat#ACC 00150) at 39°C according to the kit protocol. After incubation, the reaction mixture was loaded into the AmplifyRP® Amplicon Detection Chamber (Agdia® Inc. Cat# ADC 98800/0001) according to manufacturer’s instructions and incubated for 20 min at room temperature. Positive reactions were confirmed by the presence of both the control and test lines, and negative reactions were determined by the absence of the test line (S2 Fig).

### Statistical analysis

Graphs were prepared and all data were analyzed using GraphPad Prism 8. Statistical significance was determined by Pearson’s R and data were represented as mean ± SEM.

## Results

### Specificity and sensitivity of five different molecular and serological methods to detect *Xylella fastidiosa*

**Fig 1:**
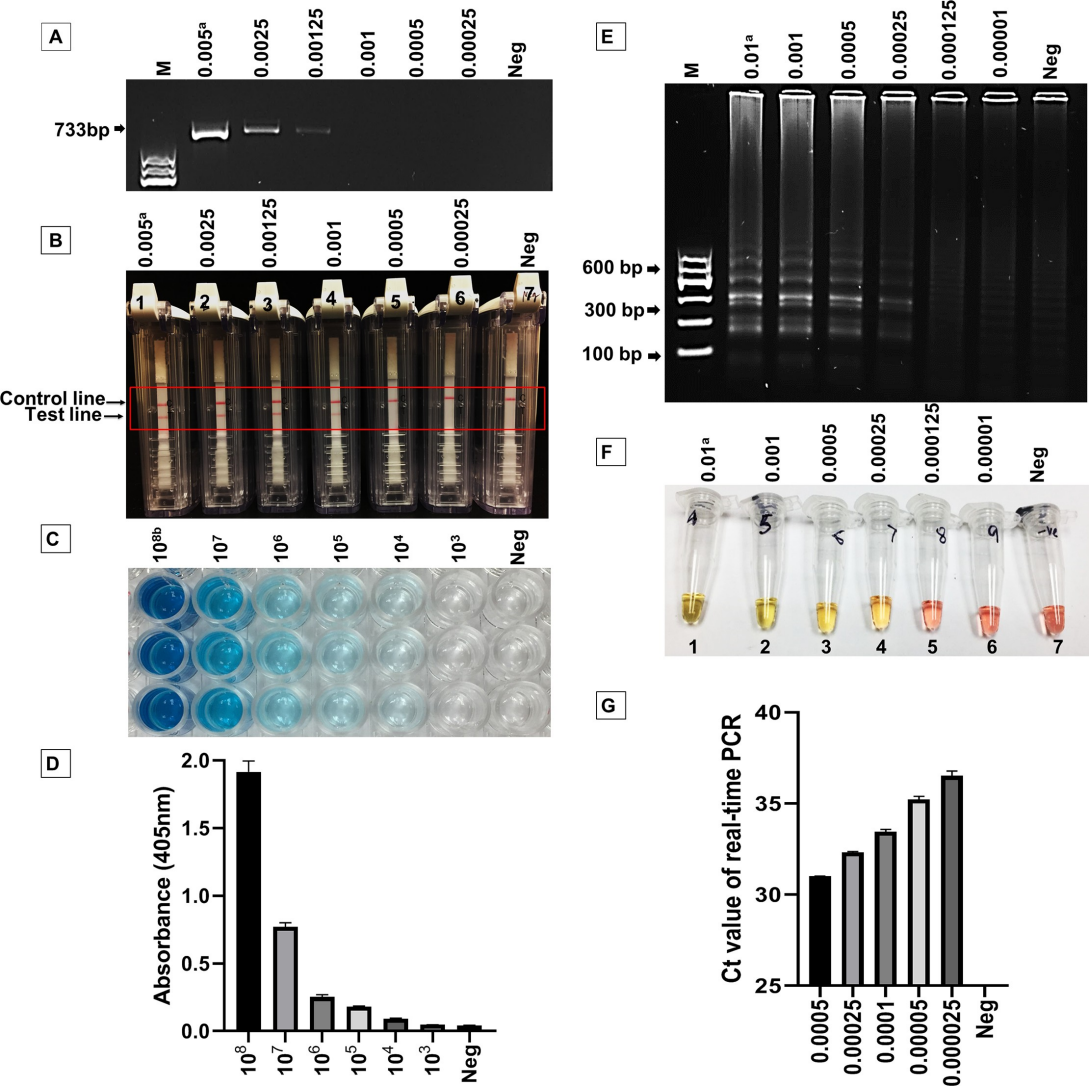
Comparative sensitivity analysis of *Xylella fastidiosa* by molecular and serological detection methods. **A**, Detection by C-PCR (conventional PCR) using RST 31/33 primers. DNA concentrations are in nanogram per microliter (ng/μl) from the serially diluted DNA extract from *X*. *fastidiosa* pure cultures. **B,** AmplifyRP® Accelar8™ positive samples were determined by the presence of test line with the control line (1 to 4) and negative samples were determined as the absence of test line (5 to 7). The concentration of viable cells were represented as cfu/ml; **C, D,** Double antibody sandwich ELISA (DAS-ELISA) samples were determined to be positive (10^5^ to 10^8^) if the absorbance at 405 nm was three times greater than the mean absorbance of control samples; **E, F,** Loop-mediated isothermal amplification (LAMP) successful amplification was visualized by agarose gel image and naked colorimetric view. Yellow color of the pH-sensitive dye Phenol Red with *X*. *fastidiosa-*positive samples (tubes 1 to 4) and pink with the negative samples (tubes 5 to 7) real-time polymerase chain reaction (qPCR); **G** Real-time PCR results. Ct = cycle threshold values.

The specificity and sensitivity of five different molecular and serological detection systems, i.e. C-PCR, real-time PCR, LAMP assay, AmplifyRP® Acceler8™ end point detection assay, and DAS-ELISA, were compared in this study. The detection limit for C-PCR for RST 31/33 (Table 1) primers was found to be 1.25 pg/μl of DNA (≈440 copies) per reaction (Fig 1A), which was less sensitive than the other molecular assays tested in this study. This finding supports the report by Harper *et al*. [26] which described the lower detection limit for C-PCR as 500 copies per reaction. However, at this marginal level, the C-PCR product in gel electrophoresis is not notably visible compared to 2.5 pg (≈875 copies) per reaction (Fig 1A). We also checked three other C-PCR primers used in earlier studies to detect *X*. *fastidiosa* (Table 1, S3 Fig). For primer set S-S-X.fas-0838-a-S-21 and S-S-X.fas-1439-a-A-19, the detection limit was similar to primers RST 31/33: 1.25pg/μl of DNA (≈440 copies) per reaction (S3 Fig). Whereas the detection limit for FXYgyr499 and RXYgyr907 primers was the lowest when compared to all other C-PCR primers used in this study (S3 Fig). Compared to all other C-PCR primers, HL5 and HL6 showed to be the most sensitive and could detect a concentration as low as 1 pg/μl of DNA (≈350 copies, S3 Fig) which supports the findings by Francis *et al*. [34] as the HL5 and HL6 primers were seen to be superior to RST 31/33 primers in detecting *X*. *fastidiosa*. However, at this low concentration, the C-PCR product was hardly visible (S3 Fig) for the primer set, suggesting that the more applicable detection limit for C-PCR is about 1.25 pg/μl of DNA (≈440 copies) per reaction for the primers used in this study. The second most sensitive molecular assay was found to be AmplifyRP® Acceler8™, which could detect a concentration as low as 1 pg/μl of DNA (≈350 copies, Fig 1B), which is also supported by a recent report where Li *et al*. [30] described AmplifyRP® Acceler8™ to be more sensitive than PCR. The next method, the real-time PCR assay, had a detection limit of 25 fg (≈9 copies, Fig 1G) per reaction but with a substantial variation between the replicate Ct values (mean Ct value 36.015 ± SEM 0.23 cycles) at this concentration, which is close to the previous findings of 10 copies per reaction of detection limit for this assay mentioned by several other reports [26,36, 37]. However, the consistent amplification was observed with a higher concentration of DNA of 250 fg or 0.25 pg (≈88 copies) per reaction, where the average Ct value was 32.32 ± SEM 0.05 cycles (Fig 1G). For the LAMP assay obtained from Harper *et al*. [26], the detection limit was found with a higher concentration of DNA. A minimum concentration of 0.25 pg DNA (≈88 copies) per reaction was required to amplify with this detection method for visualizing a color change to yellow by use of the pH-sensitive dye phenol red (Fig 1E and 1F). These results suggested that AmplifyRP® Acceler8™, LAMP and real-time PCR assays are more sensitive than the C-PCR, and that real-time PCR is the most sensitive assay among those tested for detection of *X*. *fastidiosa* DNA molecules which could detect a DNA concentration as low as 25 fg per reaction.

In comparison, the limit of detection by DAS-ELISA was 1 x 10^5^ cfu/ml based upon the absorbance at 405 nm of 0.187 ± 0.023 which was more than three times higher than the control value (0.043 ± 0.0074) that could be visualized by clear blue color development (Fig 1C and 1D). Although at 1 x 10^4^ cfu/ml reaction wells showed some indication of light blue color change, the absorbance at 405 nm was 0.093±0.013 which is three times lower than that of the control value (<3.0X) and therefore considered to be negative.

### Detection of experimental samples using C-PCR and validation with real-time PCR, LAMP, AmplifyRP® Acceler8™ and DAS-ELISA

**Fig 2:**
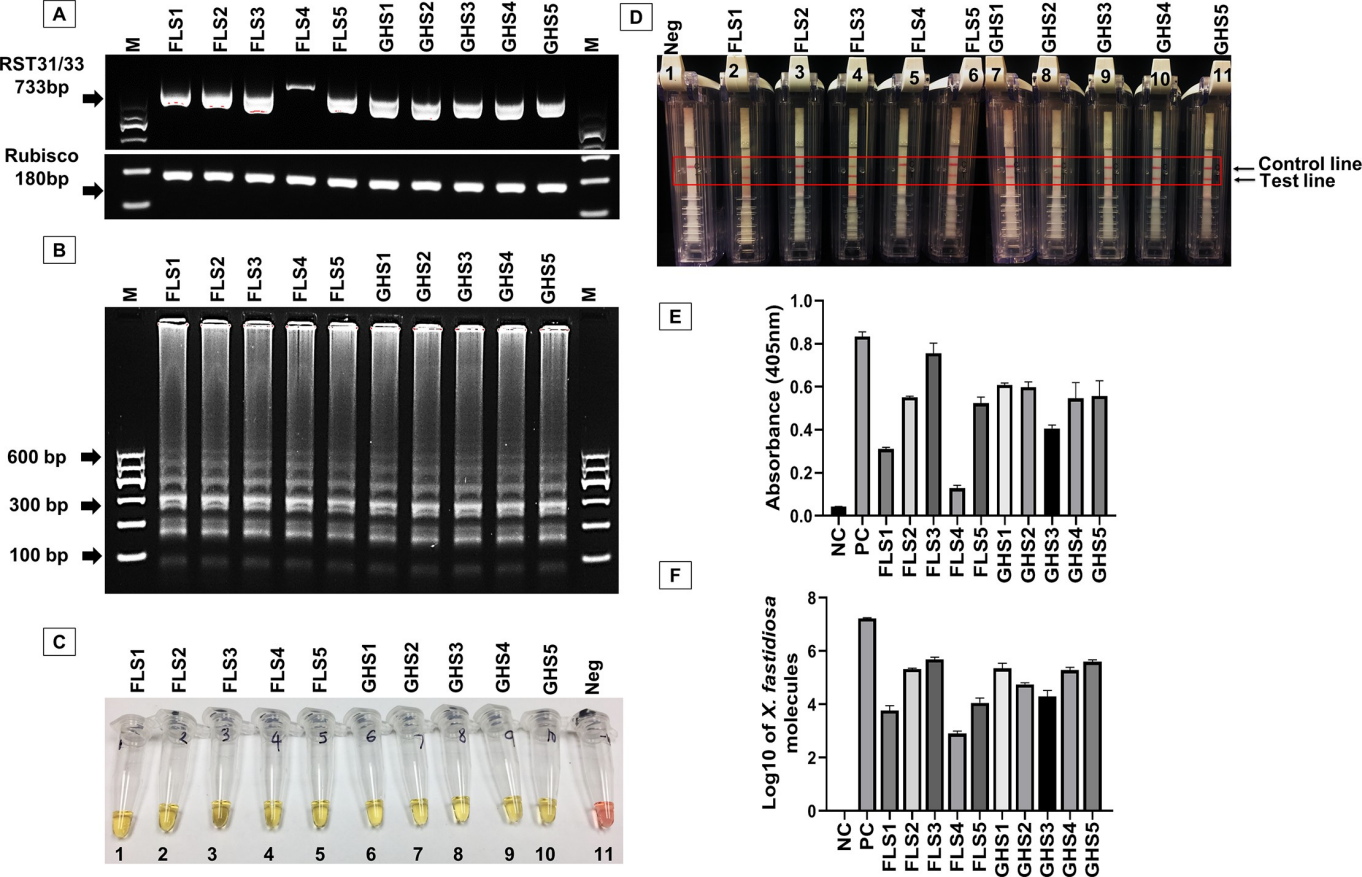
Analysis of field- and greenhouse-collected blueberry samples infected with *Xylella fastidiosa*. **A,** Amplicons obtained by C-PCR (Rubisco as internal control gene); **B,C,** LAMP successful amplification of *X*. *fastidiosa* was visualized with yellow color of the pH-sensitive dye Phenol Red (tubes 1–10) or negative sample remains pink (tube 11); **D,** AmplifyRP® Accelar8™ positive samples were determined by the presence of test line with the control line (2 to 11) and negative samples were determined as the absence of test line (1); **E,** DAS-ELISA and **F,** Real-time PCR (log10 of the copy number of *Xylella fastidiosa* molecules was obtained from the regression equation y = -3.4491x + 38.833). M = 100 bp ladder marker.

**Fig 3:**
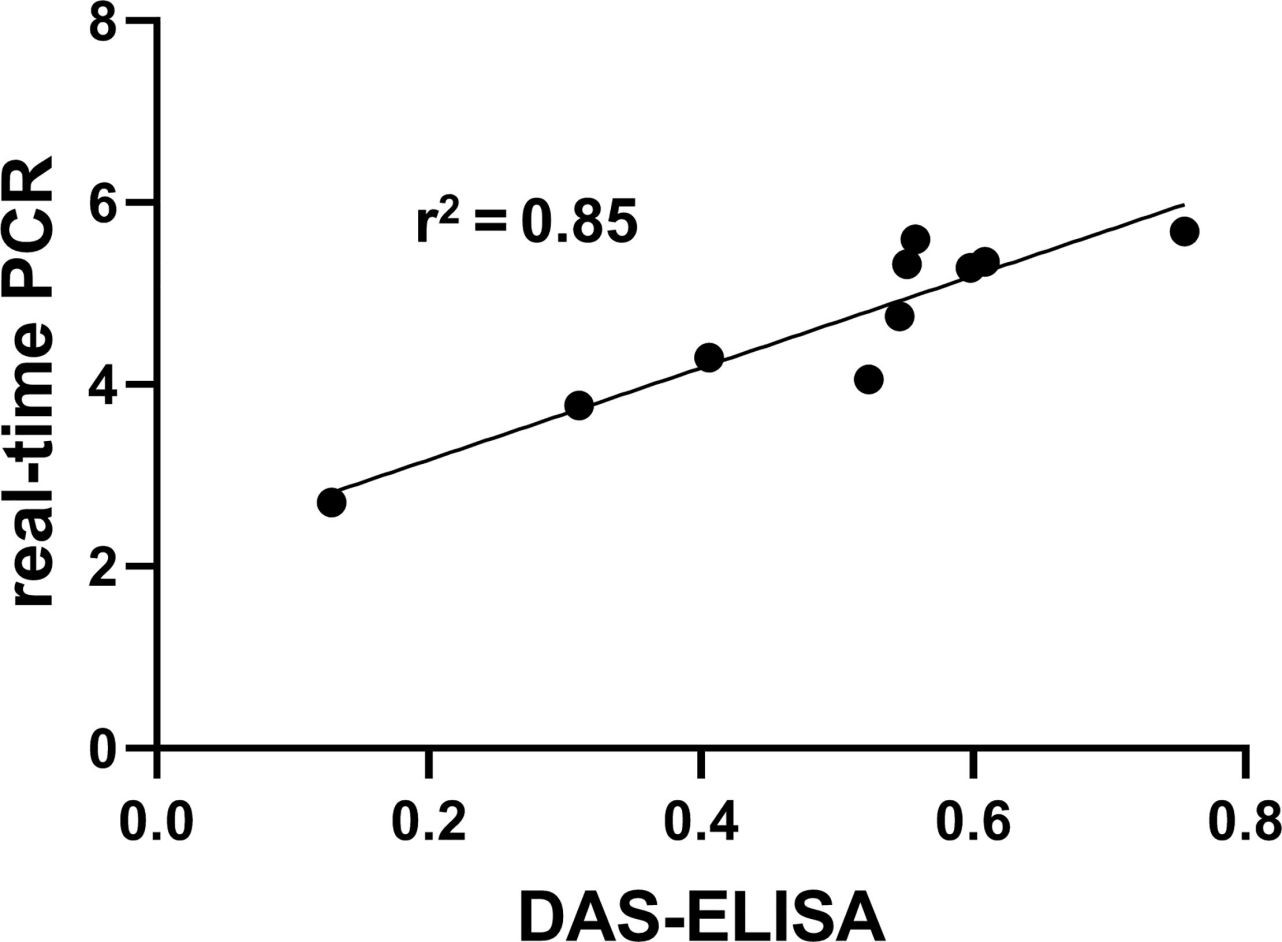
Pearson’s R^2^ correlation between DAS-ELISA and real-time PCR for field and greenhouse collected blueberry samples infected with *Xylella fastidiosa*. The R^2^ value of 0.85 indicates a high correlation between the two detection methods.

To check the detection variability among molecular and serological methods, 10 different symptomatic leaf tissue samples were obtained from the field and greenhouse and checked with C-PCR, real-time PCR, LAMP, AmplifyRP® Acceler8™ and DAS-ELISA. Among those samples, 5 were from the field and 5 from the greenhouse. Amplification of *X*. *fastidiosa* DNA was successful and variation was observed among 10 samples for RST 31/33 by C-PCR, where internal control gene Rubisco amplicons were similar (Fig 2A). C-PCR was carried out using two different cycle conditions, one with 35 cycles as described previously by Loconsole *et al*. [31] and another with 40 cycles according to Hernandez-Martinez *et al*. [38]. With the first PCR cycle conditions, sample FLS4 (field sample 4, data not shown) was not detected as positive, but could be detected with the extended cycle conditions (Fig 2A), which suggests the marginal quantity of the pathogen’s DNA molecules that are present in the sample sufficient for detection by C-PCR. Higher variation among field samples compared to greenhouse samples was also obtained by AmplifyRP® Acceler8™ assay, where the test line for the field sample number 3 (FLS3) was more prominent than the field sample number 4 (FLS4, Fig 2D). On the other hand, for the LAMP assay, all infected samples were detected successfully but without variation (Fig 2B and 2C). We also checked the amplification of the *X*. *fastidiosa* DNA for all those 10 different infected samples with real-time PCR to validate the amplification by C-PCR. The log10 of the *X*. *fastidiosa* DNA molecules present in the infected samples were calculated using the regression equation y = -3.4491x + 38.833 obtained from the standard curve prepared in this study (S2 Fig), where x is the cycle threshold. Using real-time PCR, we could amplify the *X*. *fastidiosa* 16s rRNA processing protein DNA for all infected samples where amplification for the FLS4 was lowest compared to other samples (Average Log10 value for FLS4 was 2.9 ± 0.037, Fig 2F). This validated the results obtained from C-PCR. However, the cycle threshold (Ct value) for the amplification of *X*. *fastidiosa* DNA molecules for all those infected samples didn’t exceed 30 (data not shown), reflecting higher sensitivity of the assay compared to C-PCR. Validation of the real-time PCR result was done by using the serologically-based method DAS-ELISA (Fig 2E) and by checking the correlation between the two assays using Pearson’s R (Fig 3). ELISA results from all ten samples showed a strong agreement with the outcome of the real-time PCR data and based upon blue color development (data not shown) and showed a higher correlation with real-time PCR results (R^2^ = 0.85, Fig 3). Notably, we got the lowest absorbance from DAS-ELISA for FLS4 (avg. absorbance at 405 nm for the sample was 0.162 ± 0.052) in agreement with the results from C-PCR and real-time PCR. It’s noticeable that the samples from the greenhouse used in our analysis were more similar to one another in terms of pathogen quantity than the samples from the field. Field samples had an average Log10 value for *X*. *fastidiosa* DNA molecules that ranged from 2.9 ± 0.037 to 5.68 ± 0.049 and an average OD_405_ values that ranged from 0.162 ± 0.052 to 0.76 ± 0.093, whereas greenhouse samples had an average Log10 value that ranged from 4.29 ± 0.27 to 5.59 ± 0.096 and an average OD_405_ values that ranged from 0.41 to 0.61. These results suggested that even though the detection limit varies among the evaluated techniques, the results of the several different assays showed high agreement with one another.

### Comparison of five different extant methods to detect *X*. *fastidiosa*

In this study, five different molecular and serological methods, i.e. conventional PCR or C-PCR, real-time PCR, LAMP, DAS-ELISA and RPA based end-point detection technology AmplifyRP® Acceler8 ® by Agdia® Inc. assays, were tested to compare for: 1. detection time, 2. detection limit, 3. detection cost, 4. skilled labor and lab facility needs and 5. portability. It showed that the AmplifyRP ® Acceler8 ® assay required the least time (≈42 min) to detect the pathogen in the infected samples compared with other methods, was sensitive (detection limit is ≈350 copies of *X*. *fastidiosa* g DNA molecules per reaction), and didn’t require any skilled labor, specialized laboratory facilities, or equipment and was portable for field detection (Table 2). However, the assay was the most costly on a per sample basis versus the other methods analyzed in this study (Table 2). Of course, this excludes skilled labor, specialized laboratory facility, and other instrument cost which are required by other methods. This could be a major limiting factor for this detection assay. In contrast, DAS- ELISA is least expensive but required skilled labor and laboratory facilities, was not highly sensitive (detection limit is 1x10^5^ Xf cells/ml) and needed the longest time to proceed (assay run ≈250 min, ≈536 min in total including sample and reagent preparation, Table 2). According to several reports, C-PCR is more sensitive than DAS- ELISA [39, 40], however, among the four molecular methods examined in our study, it was the least sensitive (detection limit was ≈440 copies per reaction, Table 2) and most time consuming (≈150 min to run assay).

**Table 2:** Comparison between five different molecular and serological techniques for detection of *Xylella fastidiosa*.

Detection techniques	Detection time (approx..) min.			Detection		Skilled labor need	Specialized equipment need	Lowest detection limit		Portability	Lab facility need
				Cost $^c^ (approx.)							
	Sample Prep^a^	DNA Extraction	Assay Run	DNA Extraction	Assay Run			≈ Copy per reaction (DNA or cells)	Concentration of DNA (fg)^h^		
C-PCR	3	45^b^	150	4.48^d^	1.25	Yes	Yes	440	1250	No	Yes
Real-time PCR	3	45^b^	49	4.48^d^	2.48	Yes	Yes	9	25	No	Yes
LAMP	3	45^b^	47	4.48^d^	2	No	No	88	250	Yes	No
DAS-ELISA	250	—	286	—	1.09	Yes	Yes^f^	1x10^5^^g^	—	No	Yes
AmplifyRP ® Acceler8 ®	3	5	42	14^e^	6.5	No	No	350	1000	Yes	No

a. total time in minutes required for the reagent pre-mix preparation and sample homogenization

b, d. In this study we used Qiagen™ DANeasy kit to extract DNA, other methods can be used and that might change the extraction time and cost.

c. The cost was calculated in USD considering reagent needs excluding sample preparation, specialized lab instruments like thermo-cycler or heat-block and skilled labor cost

e. Cost per sample processing using AmplifyRP® XRT+ for Xf, Cat# XCS 34501/0048

f. spectrophotometer is needed to get the absorbance at OD405nm

g. *Xylella fastidiosa* cells/ml

h. fg = femtogram.

Although, considering per sample cost and accessibility, C-PCR is still considered as an important tool for molecular based diagnosis [31,34]. On the other hand, real-time PCR was the most sensitive assay for detection of *X*. *fastidiosa* DNA molecules (detection limit is ≈9 copies of DNA per reaction), and required less time to carry out (≈49 min), but is not portable to the field and necessitated skilled labor, specialized lab facilities and a thermocycler (Table 2). LAMP assay was less sensitive compared to real-time PCR (detection limit is ≈88 copies per reaction), but was less costly per sample, relatively quickly performed (≈47 min), showed greater sensitivity than C-PCR, was portable and didn’t require any specialized thermo-cycler, laboratory facilities or skilled labor to carry out (Table 2).

In conclusion, although *X*. *fastidiosa* was successfully detected from infected blueberry samples using all of the five different molecular and serological assays, real-time PCR was the most sensitive and reliable assay to detect the pathogen in laboratory conditions based upon pathogen detection quantity determinations. On the other hand, considering the portability, sensitivity, cost-effectiveness(including laboratory facility and skilled human labor cost) and robustness, LAMP and the RPA based end-point-detection technology AmplifyRP® Acceler8 ® were more convenient and can be widely used for on-site detection of the pathogen.

## Discussion

It is important to select the appropriate diagnostic tool to detect the pathogen in most reliable manner. From a diagnostic utility standpoint, the benefits of any technique to detect causal agent of diseases depends on the simplicity, specificity, sensitivity, robustness, cost-effectiveness and suitability of that tool under any circumstances. *X*. *fastidiosa* was selected as a target organism for detection from infected blueberry samples, since it has become a major threat to blueberry production in Georgia and in the other parts of North America [2, 4–10]. There are several reports where the detection of *X*. *fastidiosa* has been compared using different molecular and serological techniques [19, 23–26, 31, 34, 38, 41, 42]. Among these, some studies described the development of a rapid, reliable and superior diagnostic tool on SYBR ® Green-based or TaqMan™ based real-time PCR assay compared to other conventional universal qPCR targeting the bacterial conserved region to detect the pathogen [23, 25, 26,34]; however, these tools are more applicable for laboratory-based detection rather than field or on-site detection. There are several studies of on-site detection of pathogens using real-time PCR detection technology [43–46] including *Phytophthora ramorum* [43], *X*. *fastidiosa* [45], white spot syndrome virus (WSSV) and infectious myonecrosis virus [46], but these required an expensive, portable, and specialized thermocycler. On the other hand, the LAMP assay is not as sensitive as real-time PCR, but is highly specific and more sensitive than C-PCR and AmplifyRP® Acceler8 ® and could easily be used with colorimetric reaction for on-site detection of pathogens [30, 47]. Besides those studies, a thorough comparison in terms of sensitivity, robustness and cost-effectiveness between molecular and cellular techniques and their applicability from laboratory to field detection has not been compared. In one study, Harper *et al*. [26] compared three different techniques and developed a superior real-time PCR and LAMP assay targeting 16s rRNA processing protein rim (XF_0108) which was more sensitive than the assays described by Francis *et al* [34]. In our study, we did a comparison between five different diagnostic methods to understand laboratory and field applicability of those techniques. In contrast to Harper *et al*. [26], we showed the detection limit for PCR based and PCR derivatives techniques, i.e. C-PCR, real-time PCR, LAMP assay and RPA based detection techniques i.e. AmplifyRP® Acceler8®, not only based on copy number but also based on DNA concentration of the *X*. *fastidiosa* (Fig 1, Table 2). In addition to the molecular techniques, we also tested the serological based detection technique DAS-ELISA to get the detection limit for the assay. The C-PCR assay was conducted in this study as it remains a common baseline detection technique for several different countries [25, 26]. The detection limit for *X*. *fastidiosa* specific C-PCR was compared four different primer sets used in this study (Table 1), and ranged from 2.5 pg to nearly 1.0 pg DNA/ reaction (Fig 1, S3 Fig). In addition, there was some minor variation between C-PCR detection levels which supports a previous report by Nissen [48] where variation among *X*. *fastidiosa* specific C-PCR primers detection limit ranged from 2.5 pg to less than 1.0 pg DNA/ reaction, but we achieved an approximate detection limit for this assay for the primers we used in this study. On the other hand, the limit of detection for DAS-ELISA was 1 x 10^5^ cfu/ml. Here, we didn’t calculate the cell numbers required for DNA extraction used for molecular-based detection study, but previously Minsavage *et al*. [39] observed the 100X higher sensitivity of C-PCR over the DAS-ELISA assay for *X*. *fastidiosa* detection, where the limit of detection was 1X10^2^ cfu per reaction for PCR and 2X10^4^ for ELISA. In the study by Nissen, it was stated that the lowest detection limit for PCR based detection by RST 31/33 primers was 80 cfu of the pathogen, although at this low level the PCR product was nearly invisible and was more clear at the cfu count 8x10^2^ [48]. An additional experiment with the DNA sample extracted from a pure culture of a known concentration of *X*. *fastidiosa* would give a better idea about the detection sensitivity between C-PCR and ELISA.

In this study, the real-time PCR could detect the least amount of *X*. *fastidiosa* DNA (detection limit 25 fg, ≈9 copies, Fig 1, Table 2) compared to other assays; followed by LAMP (detection limit 0.25 pg, ≈88 copies, Fig 1, Table 2), AmplifyRP® Acceler8 ® (detection limit 1 pg/μl, ≈350 copies, Fig 1, Table 2) and C-PCR (detection limit 1.25 pg, ≈440 copies, Fig 1, Table 2) which supports the data from Harper *et al*. [26], where real-time PCR was the most sensitive assay after LAMP and C-PCR. Besides the detection limit comparison of the assays, the compatibility of 4 different molecular and 1 serological assays using naturally infected samples from the field and artificially inoculated samples from greenhouse shown all of those assays were able to detect the pathogen from infected samples. There was a higher but less than 1 correlation (R^2^ = 0.85, R^2^ <1, Fig 3) between real-time PCR and DAS-ELISA assay results, which reflects the idea about sensitivity limit variation between the two different techniques. For LAMP, we did not see any difference between samples because of the abundant gDNA of *X*. *fastidiosa* molecules present in the sample, which was markedly higher than the detection limit by the assay (Fig 2).

We extracted DNA in a laboratory facility and to test for on-site detection of pathogens by LAMP used Pickpen (Pickpen, Bio-nobile) described by Schaad *et al* [45]. We compared the yield from both extractions where on-site DNA extraction gave a lower amount of DNA than laboratory-based DNA extraction (data not shown), which caused false-negative result for the sample with a marginal titer (data not shown) supports the idea of Harper *et al* [26]. On the other hand, recombinase-polymerase amplification (RPA) based end-product detection technology AmplifyRP ® Acceler8® (Agdia® Inc.) has a simplified extraction procedure which gives a clear advantage of this method above all other methods studied here. In several studies, it was stated that AmplifyRP ® Acceler8 ® is more sensitive than C-PCR [30, 49]. We also observed higher sensitivity for AmplifyRP® Acceler8 ® than C-PCR, but it was less sensitive than the LAMP (Fig 1, Table 2) in our analysis. Both the LAMP and AmplifyRP ® Acceler8 ® (Agdia® Inc.) end-product detection assays were rapid (≈42 to 47 min), did not require any skilled labor or specialized instruments, and were sensitive enough to detect on-site pathogens (i.e. portable in the field). It should be noted that in one recent study by Karakkat *et al*. [50] that used the AmplifyRP® Acceler8® for turfgrass pathogen detection, it had a lower false-positive reaction rate compared to LAMP assays and was more convenient for field or diagnostic laboratory application. But considering cost, LAMP assay would be a superior choice–a factor which is limiting for AmplifyRP® Acceler8 ® end-product detection assay. On the other hand, real-time PCR was found to be the most sensitive method in this study compared to other assays (could detect as low as ≈9 copies per sample), was less costly compared to AmplifyRP ® Acceler8 ® (Table 2), and relatively fast (≈49 min) for detection and quantification of *X*. *fastidiosa* present in the sample. Furthermore, real-time PCR can even differentiate between subspecies and genotypes [25,51–53], although it does require laboratory facilities, an expensive thermocycler and skilled labor to proceed. The other two assays, C-PCR and DAS-ELISA, though they required a longer times to carry out, are the least costly and are still used for baseline detection of the pathogen in laboratory condition for large numbers of samples (Table 2). In conclusion, comparing all five methods suggests that LAMP and AmplifyRP ® Acceler8 ® are suitable for on-site detection of the pathogen, but real-time PCR is unique in its ability to detect, quantify, and study a pathogen in a well-equipped laboratory condition.

## Supporting information

S1 Fig Real-time polymerase chain reaction (qPCR) standard regression curve of the log of the amounts of *Xylella fastidiosa* DNA versus the corresponding cycle threshold (Ct) values. Each value is the mean of three separate runs of the qPCR assay. The efficiency (E) of the qPCR is given as [10(−1/slope)-1].
(TIF)

S2 Fig An example photograph of the detection of *Xylella fastidiosa* from uninfected and infected blueberry samples using AmplifyRP® Acceler8™. The control and test lines are indicated by arrows. The *Xylella fastidiosa* negative and positive samples are indicated by—and + signs respectively.
(TIF)

S3 Fig Comparative sensitivity analysis of *Xylella fastidiosa* detection by C-PCR (conventional PCR) using S-S-X.fas, FXYgyr, and HL5/6 primers. DNA concentration is in nanogram per microliter (ng/μl) from the serially diluted DNA extracted from *X*. *fastidiosa* pure cultures. The black arrowhead shows the PCR product size in base pair.
(TIF)
