# Supplementary figures and images for: Comparative analysis of different molecular and serological methods for detection of Xylella fastidiosa in blueberry
Source: PLoS One. 2019 Sep 3;14(9):e0221903. doi: 10.1371/journal.pone.0221903 (PMC6719857; doi:10.1371/journal.pone.0221903)

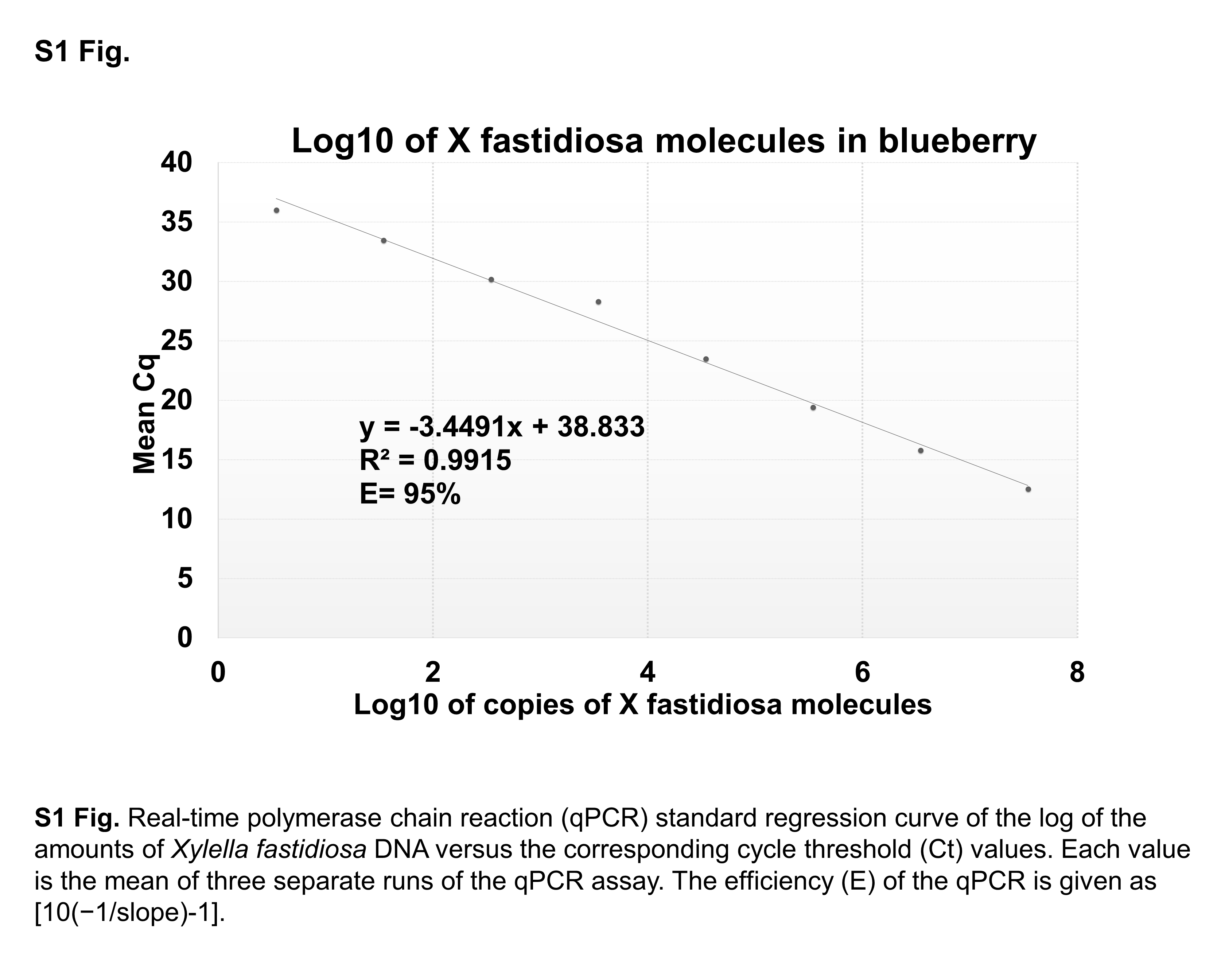

Supplement: S1 Fig — Each value is the mean of three separate runs of the qPCR assay. The efficiency (E) of the qPCR is given as [10(−1/slope)-1]. (TIF) [file pone.0221903.s001.tif]

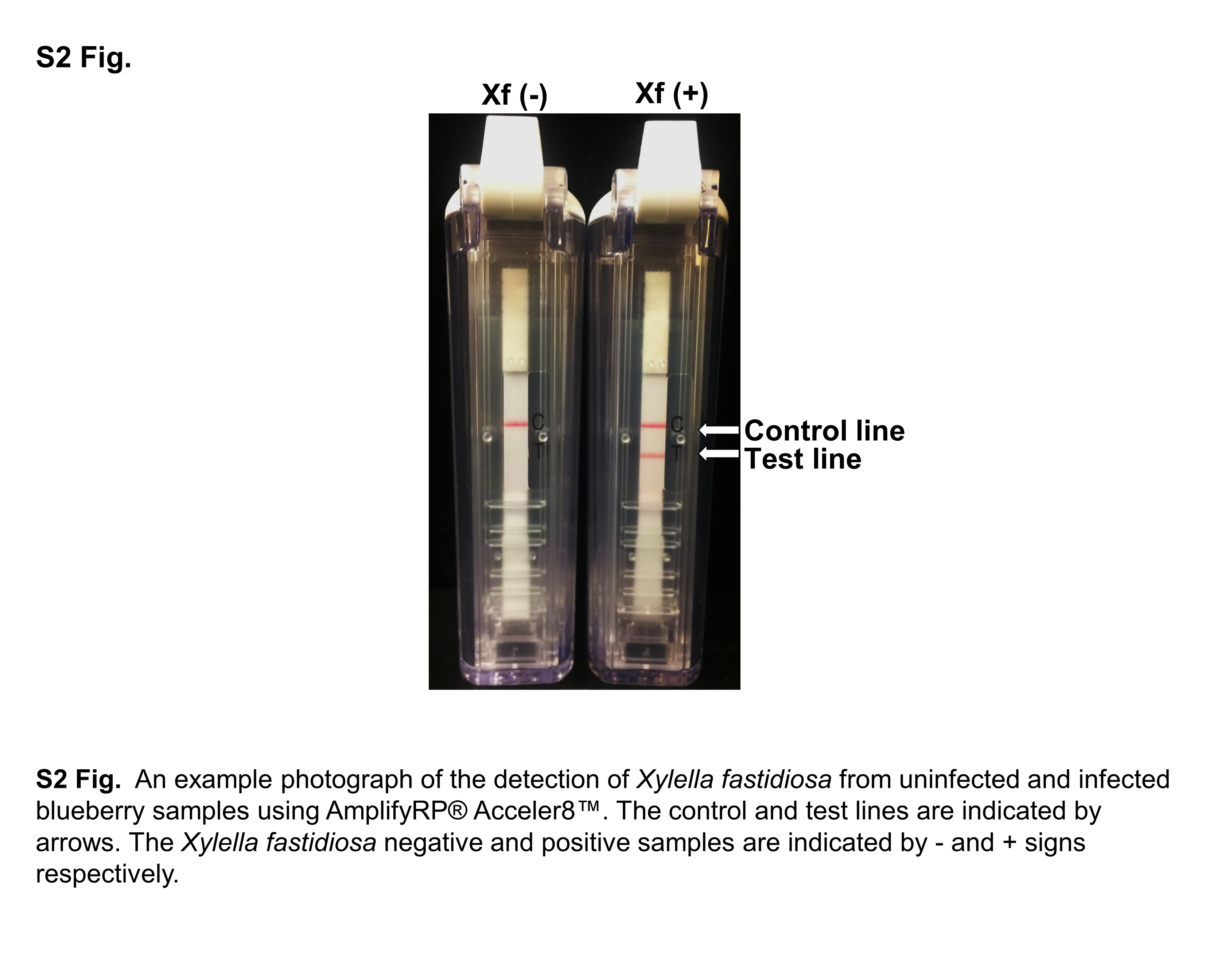

Supplement: S2 Fig — The control and test lines are indicated by arrows. The Xylella fastidiosa negative and positive samples are indicated by—and + signs respectively. (TIF) [file pone.0221903.s002.tif]

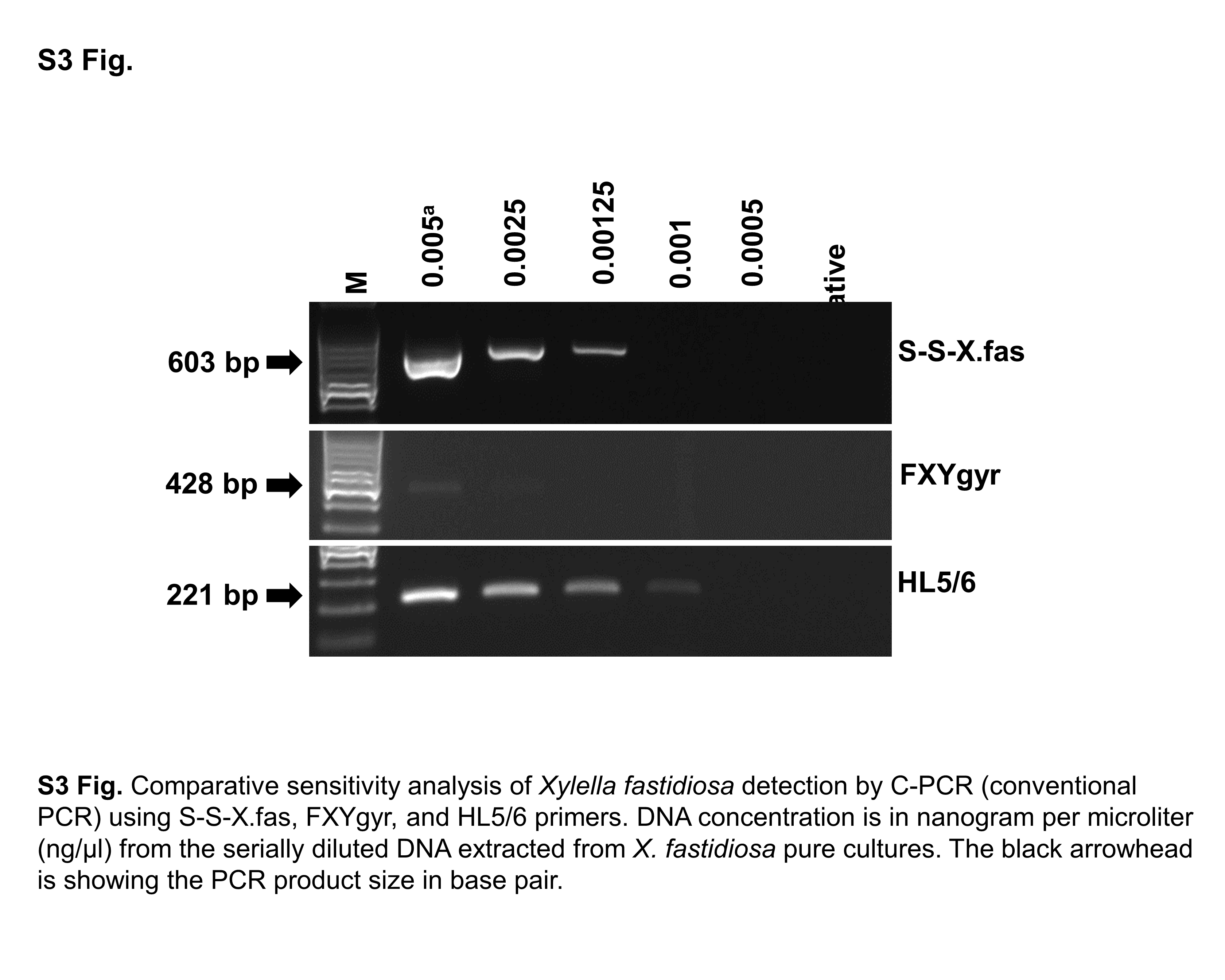

Supplement: S3 Fig — DNA concentration is in nanogram per microliter (ng/μl) from the serially diluted DNA extracted from X. fastidiosa pure cultures. The black arrowhead shows the PCR product size in base pair. (TIF) [file pone.0221903.s003.tif]
